# The Role of Expectation and Beliefs on the Effects of Non-Invasive Brain Stimulation

**DOI:** 10.3390/brainsci11111526

**Published:** 2021-11-18

**Authors:** Miriam Braga, Diletta Barbiani, Mehran Emadi Andani, Bernardo Villa-Sánchez, Michele Tinazzi, Mirta Fiorio

**Affiliations:** 1 Department of Neurosciences, Biomedicine and Movement Sciences, University of Verona, 37131 Verona, Italy; miriam.braga@univr.it (M.B.); diletta.barbiani@univr.it (D.B.); mehran.emadiandani@univr.it (M.E.A.); michele.tinazzi@univr.it (M.T.); 2 Department of General Psychology, University of Padova, 35131 Padova, Italy; 3 Center for Mind/Brain Sciences (CIMeC), University of Trento, 38068 Rovereto, Italy; b.villasanchez@unitn.it

**Keywords:** non-invasive brain stimulation, transcranial magnetic stimulation, transcranial direct current stimulation, placebo effect, nocebo effect, expectation

## Abstract

Non-invasive brain stimulation (NIBS) techniques are used in clinical and cognitive neuroscience to induce a mild magnetic or electric field in the brain to modulate behavior and cortical activation. Despite the great body of literature demonstrating promising results, unexpected or even paradoxical outcomes are sometimes observed. This might be due either to technical and methodological issues (e.g., stimulation parameters, stimulated brain area), or to participants’ expectations and beliefs before and during the stimulation sessions. In this narrative review, we present some studies showing that placebo and nocebo effects, associated with positive and negative expectations, respectively, could be present in NIBS trials, both in experimental and in clinical settings. The lack of systematic evaluation of subjective expectations and beliefs before and after stimulation could represent a caveat that overshadows the potential contribution of placebo and nocebo effects in the outcome of NIBS trials.

## 1. Introduction

### 1.1. Non-Invasive Brain Stimulation and Variability in Experimental Data

In the last two decades, non-invasive brain (NIBS) stimulation techniques have been extensively applied in clinical and cognitive neuroscience, making a significant contribution to a better understanding of the neurophysiological correlates of several cognitive functions. NIBS comprises of different techniques based on magnetic or electrical stimulation of the scalp. Transcranial magnetic stimulation (TMS) consists of a transient magnetic field applied on the scalp through a coil, inducing in turn a transitory electric current in the brain. The magnetic pulse induces a rapid depolarization of the cell membranes under the coil [1,2], followed by depolarization or hyperpolarization of other neural populations, i.e., TMS directly elicits action potentials in the stimulated neurons. TMS is used as a therapeutic aid to treat patients with neurological or psychiatric disorders [3,4,5,6], as well as for experimental purposes [5]. TMS can be applied as one stimulus at a time (single pulse), as trains of stimuli delivered at a fixed frequency, usually of 1–20 Hz (repetitive TMS), or in trains combining different frequencies (i.e., 50 Hz pulse trains repeated at a rate of 5 Hz), described as theta burst stimulation (TBS). Among TBS approaches, intermittent theta-burst stimulation (iTBS) produces a persisting increase in the amplitude of motor responses evoked by TMS, whereas continuous theta-burst stimulation (cTBS) leads to suppression of TMS evoked responses [7].

In contrast, transcranial electrical stimulation techniques (tES), such as transcranial direct current stimulation (tDCS), transcranial random noise stimulation (tRNS), and transcranial alternating current stimulation (tACS), are neuromodulation tools, in which a weak electrical current is applied on the scalp through two or more electrodes [8,9,10,11]. Among tES, tDCS is the most widely used and studied and, therefore, this review focuses on tDCS only. tDCS induces a subthreshold polarization of cortical neurons and acts by changing neuronal excitability, by inducing modifications in the resting membrane potentials, and in the postsynaptic activity of the stimulated neurons, without directly affecting action potentials. tDCS can alter the spontaneous firing rate, leading to changes in synaptic activity [12,13,14,15]. When the anode is positioned over the cortical site of interest and the cathode is positioned over a reference point (either cephalic or extra-cephalic), a depolarization of the resting membrane potential is induced in the stimulated brain area, together with an increase in neuronal excitability and firing rate. Conversely, when the cathode is positioned over the cortical site of interest and the anode over a reference point, a hyperpolarization and a decrease in neuronal excitability is induced in the stimulated brain area [8,9]. The former approach is known as “anodal stimulation” and the latter as “cathodal stimulation”. The reported polarity-dependent effects are, however, not consistent, being mainly described in the motor domain and to a lesser extent in cognitive investigations [16].

The effects of both TMS and tDCS on the brain and on motor and cognitive functions may depend on a variety of characteristics, such as position of the coil or electrodes, direction and intensity of the current (and also frequency and duration in the case of tDCS) [9,17,18], properties of the stimulated brain tissue [19], demographic variables of the stimulated individual (e.g., gender and age) [20,21,22], and the cognitive state of the stimulated brain area [23,24]. All these conditions have been proposed as explanations for the inconsistencies in results found across studies. In 2013, Miniussi and colleagues proposed a unified model which posited that the effects of NIBS are linked to noise induction, which in turn interacts with several parameters, such as the characteristics of the stimulation and the task performed during the stimulation [25]. More specifically, the authors reasoned that the final response to a target stimulus does not depend solely on the strength of the signal induced by the target itself but depends also on the ratio between the signal and other irrelevant activity, namely, the noise [25]. Thus, successful performance in behavioral tasks depends on the relation between the signal (i.e., neurons coding for the target in a particular task) and the noise (i.e., neurons whose activity is non-specific for the task at hand). This hypothesis is strongly linked to so-called state dependency that refers to the state of the system at the time at which the stimulation is applied (TMS or tDCS). It has been shown that the effects of TMS are proportional to the level of neuronal activation during the application of the pulses [26]. In the case of tDCS, the stimulation does not directly induce action potentials, but modulates the neuronal response threshold, facilitating the neural activation of all neurons, even those not involved in the task. This could result in an increase not only of the signal, but also of the noise. Consequently, the effect of the stimulation will be highly influenced by the pre-existing state of the system because its effect depends on the activity of the stimulated area.

In this narrative review, we propose that participants’ expectations present before or during the stimulation session could have a role in shaping the effect of NIBS on the brain and on behavior, thus partially accounting for the inconsistencies found in the literature. In other words, we speculate that participants’ beliefs about the type of stimulation received, or their expectations and prior beliefs about the effects of the stimulation on the brain and on performance, could represent a pre-existing state that influences the effects of the stimulation itself. With a change of emphasis from the past, recent research is devoting increasing attention to the evaluation of participants’ expectations and subjective feelings with respect to the experiment in general and to the effects of the stimulation in particular. Here, we adduce some evidence showing that individual expectations and placebo/nocebo-like mechanisms could have a role in explaining the variability of NIBS outcomes. To this end, we performed a literature search of the PubMed and Scopus electronic databases, using the terms (“NIBS” OR “TMS” OR “tDCS”) AND (“Placebo” OR “Nocebo” OR “Expectations” OR “Blinding”). Additional articles were found from supplementary sources, such as *Google Scholar*. Full-text articles were screened against the following predefined inclusion criteria: English language, experimental studies (either clinical populations or healthy volunteers), systematic reviews and meta-analyses, published up to October 2021. Based on these criteria, we selected 30 studies (18 with TMS and 12 with tDCS). Details related to each single study (e.g., stimulation intensity, site of stimulation, methods, etc.) are presented in Table 1 and Table 2.

### 1.2. Placebo and Nocebo Effects

Expectations can be defined as the belief in the likelihood of something happening and as such, they can modulate a variety of cognitive processes, such as perception [57], motor control [58], and working memory [59], among others [60]. This aligns with response expectancy theory [61], whereby non-volitional responses (e.g., pain perception, emotional reactions, and other subjective stress and sensations) can be elicited or enhanced by the expectancy of their occurrence, with a tendency to be self-confirming. The expectancy theory provides a theoretical framework to explain the crucial role of contextual variables in inducing placebo/nocebo effects. These effects may be viewed as emblematic phenomena demonstrating how explicit or implicit contextual cues may boost expectations, leading to changes in perception, behavior, and physiology. Specifically, the outcome following placebo administration is not ascribable to the intrinsic therapeutic properties of the (inert) treatment but to verbal suggestions, rituals, and symbols surrounding the therapy [62]. If “coated with” positive meaning, these elements have been found to induce positive expectations of symptom improvement, which can lead to an actual clinical amelioration, at both subjective and objective levels. For example, positive verbal suggestions alone may be sufficient to reduce pain perception and pain sensation [63], and modulate motor [64] and cognitive performance [65]. Furthermore, verbal suggestions may affect anxiety, which has been shown to worsen symptoms [66]. Indeed, the opposite counterpart of the placebo effect, i.e., the nocebo effect, refers to a worsening in symptoms following the administration of an inert treatment along with negative verbal suggestions or cues of symptom worsening [67].

In inducing the placebo effect, learning mechanisms are also strongly involved, especially preconditioning procedures with real drugs [68]. In the case of pharmacological preconditioning, a repeated exposure to an active agent that is subsequently replaced by a placebo leads to substantial placebo responses that mimic the effects of the active agent. In other words, the experience of benefit due to the exposure to a drug becomes beneficial when the drug is later substituted by a similar yet inert (placebo) treatment. Research has increasingly shown how this type of conditioning paradigm may be effective in boosting the placebo response even in clinical conditions, such as Parkinson’s disease [69]. This is in concert with evidence reporting greater placebo effects when participants experienced an effective conditioning procedure with an analgesic treatment, compared to those who had previously experienced an ineffective analgesic treatment, suggesting how the quality of prior experience may be a crucial factor in shaping the magnitude of the placebo response [70]. Apart from the quality of the treatment or procedure employed during conditioning, the quantity, i.e., the length, of the exposure has also been found to modulate outcome [71]. Finally, placebo and nocebo effects can be induced via observational learning, that is, by simply observing another person’s behavior [72,73].

Overall, placebo and nocebo effects may be considered an example of how sensory information may be “overweighted” by prior beliefs, which represents a common phenomenon in predictive coding networks. According to this viewpoint, our brain is an inference machine that is actively engaged in the prediction and explanation of its sensations, and seeks confirmation, by using probabilities, and relying on previous models or hypotheses (‘priors’), to infer the state of the world [74]. Insights about the salient features of model-based computations in the prediction of future outcomes derive from studies showing that the magnitude of medio-frontal negativity tracks the timing of salient events, with higher amplitude for outcomes occurring at unexpected times [75]. This observation is consistent with the predicted response-outcomes (PRO) model that posits that the medial prefrontal cortex (mPFC) is critically involved in learning predictions of future outcomes, concerning not only their likelihood of occurrence but also their timing [76]. Model-based computations have been found to be crucial also in other domains, for example in the acquisition of fear conditioning, again involving the mPFC [77].

In general, expectations and learning may act as a priori stimuli (‘priors’), which may hijack perception in order to reduce prediction errors, i.e., when there is mismatch between predictions and incoming sensory information, in order to reduce “surprise” and maintain a more stable and foreseeable representation of the world [78].

The extent to which these priors may bias perception depends upon their level of precision (strength or salience), but also on the precision of the incoming sensory inputs. For instance, when a sensory input is weak, noisy, or ambiguous, expectations prevail and can strongly bias perception [79,80]. Conversely, when stimuli are non-ambiguous and predictions are valid, expectations do not strongly bias perception, but rather can exert their effects by allowing rapid detection of stimuli [81,82] and enhanced accuracy [83]. In addition, prior knowledge modulates perception when it is reliable and stimuli are ambiguous, while it has less effect when stimuli are reliable and expectations are poorly precise [57]. Taken together, these findings suggest how prior beliefs and expectancies are crucial factors that cannot be neglected when considering the overall effect of a given treatment or procedure.

## 2. The Need of Blinding in Sham Protocols

### 2.1. Sham Stimulation: Definition and Open Issues

Classically, participants’ thoughts, beliefs, and expectations about NIBS have been conceptualized as confounding factors to be controlled for in order to unravel the effect of active stimulation. Hence, the development of NIBS techniques has required the implementation of effective control procedures, to rule out any non-specific effects due to acoustic or tactile sensations experienced by the participants during the stimulation, and to avoid unblinding. To achieve these aims, sham stimulation protocols have been developed to mimic the sensations experienced by participants during active stimulation, without substantially stimulating the brain. It should be noted that whereas these procedures could control for participants’ specific beliefs on the type of stimulation received, they do not necessarily control for more general prior beliefs or expectations about the effects of the stimulation on the brain and on performance. Here, we discuss the blinding issues related to the first aspect, leaving the description of the second aspect to subsequent paragraphs.

Regarding TMS, several sham protocols have been proposed [84]. Generally, sham TMS is applied by changing the position of the coil. However, this procedure does not ensure complete exclusion of residual brain stimulation [84,85]. To overcome this issue, it is possible to use a regular coil turned upside-down to reduce active brain stimulation. A second approach makes use of purpose-built sham TMS coils that resemble regular coils but attenuate the magnetic field. In this case, the sham coil can be positioned precisely over the target brain area and produces the same auditory sensations induced by active stimulation. Finally, realistic sham TMS coils producing an auditory click and administering a weak electrical stimulation to reproduce the skin sensation have also been used [86].

Although still controversial, it has been proposed that sham TMS per se could have direct neuromodulatory effects on the brain, as evidenced by changes in the EEG phase connectivity and in somatosensory evoked potentials amplitude induced by sham cTBS over the DLPFC [87], as well as by the weak electric field induced in the left frontal cortex by sham TMS [88].

We would like to briefly acknowledge that sham TMS could influence behavior through other mechanisms, for instance by capturing participants’ attention. With respect to this, Duecker and Sack [27] (Table 1) showed that sham TMS lateralized to one or the other hemisphere caused an automatic shift of attention toward the position of the coil, thereby facilitating target detection in the corresponding hemifield. Hence, the sensory side effects of TMS could influence performance depending on the location which the sound of the TMS pulse stems from [27,28,89].

Even for tDCS, sham stimulation has the goal of inducing in the participants the same sensations experienced with the real stimulation, without changing underlying neural activity. As a result, sham procedures are crucial for demonstrating the effects of real stimulation in experimental and clinical domains. However, inconsistencies in the literature are present regarding the modulatory effects of tDCS [90], reporting paradoxical effects or lack of modulation, which could be due to issues related to sham blinding rather than to failure in active tDCS protocols.

Usually, sham stimulation for tDCS is applied for a few seconds at the beginning of the stimulation session, or with a constant current at low intensity for the entire duration of the session [91,92,93]. The aim of these sham stimulation protocols is to let participants feel a sensation on the skin, equivalent to that felt during active stimulation. As shown for sham TMS, we briefly mention here that sham tDCS could also potentially exert some indirect neurobiological effects [91]. For instance, a study by Nikolin et al. [93] evaluated the behavioral and neurophysiological effects of bilateral tDCS administered over the DLPFC on a working memory task by comparing different active tDCS and sham current intensities (2 mA and 1 mA for active tDCS, 0.034 mA, 0.016 mA, and 0 mA for sham). Results indicated that, although no influence was found on performance at the working memory task, a single session of sham tDCS could exert neuromodulatory effects at an intensity of 0.034 mA. These results suggest that a sham protocol previously considered as inactive (0.034 mA) may potentially induce physiological effects. This is consistent with the literature reporting possible neural effects of micro-ampere-scale currents [15] and proposing that small amounts of noise added to the system might induce an enhancement in activation (so-called stochastic resonance [94]). Finally, although one study found an increase in MEP amplitude after sham tDCS [95], a systematic review reported no effect of sham stimulation on corticospinal excitability, leaving this issue open for deeper investigation [96].

### 2.2. Blinding Success and Failure in NIBS Studies

Recent work by Flanagan et al. [29] investigated whether active intermittent theta burst stimulation (iTBS), which is a type of repetitive TMS, could be distinguished from sham TMS with regards to visual, acoustic, and tactile sensations. After one hour of having received either active or sham iTBS, participants were required to judge the type of stimulation. The results showed that when participants underwent real iTBS, they could predict the type of stimulation at chance level (55% of participants), whereas when they underwent sham stimulation, they correctly predicted the type of stimulation above chance (74% correctly reported having received sham stimulation). Moreover, the percentage of correct responses was higher on the second visit compared to the first one, hinting at a role of previous experience in recognizing the type of stimulation (Table 1).

As with TMS, tES posits the critical issue of blindness efficacy. For example, Turi et al. [30] suggested that the amount of discomfort felt during active tDCS (delivered at 1 mA for 20 min) can break blinding, since participants reported more discomfort after real as compared to sham tDCS and correctly “guessed” the experimental condition (sham vs. active) above chance level. Importantly, none of the participants had any prior experience with tDCS and each participant took part in only one condition, meaning that they could not have a reference point to compare sensations to [30] (Table 1).

In another study, Greinacher et al. [31] assessed the time course of sham-blinding by measuring every 30 s whether participants could correctly identify active or sham tDCS (in which the current lasted only 20 s at the beginning of the session) and how confident they were about their decision. Results showed that participants were aware that the duration of active anodal tDCS was longer than the duration of sham stimulation. More importantly, by analyzing more deeply the time-course of the failed sham-blinding protocol, it was observed that participants were confident that the stimulator had been deactivated as soon as the stimulation stopped (Table 1). The limitation of this procedure is that by continuously asking participants whether they think they are (or were) being stimulated or not, one could induce them to focus their attention on the sensations felt during the stimulation rather than on the task at hand. Nonetheless, these results are consistent with previous work positing that at a higher intensity of stimulation (e.g., 2 mA), sham blinding is inefficient [32] (Table 1). All these observations raise concerns about the procedures typically used for sham tDCS and should encourage the scientific community to improve blinding procedure in tDCS research to increase reproducibility and avoid experimental biases. Fonteneau et al. [91] proposed multi-electrodes montage and “active control” (i.e., stimulating a brain region considered to be inactive in the task at hand) as useful strategies to avoid sham confounding effects in tDCS trials.

The impact of blinding issues in tDCS research has also emerged in the clinical domain. For example, a study by Brunoni et al. [33] showed that 83% of the participants with major depression correctly guessed when they received active tDCS and 37% correctly guessed when they received sham tDCS. Importantly, participants who correctly guessed whether active or sham tDCS was administered had greater clinical improvement in depression symptoms [33] (Table 1). This study underlines that, if not properly controlled, beliefs about the type of stimulation received could have a decisive role in determining clinical outcomes.

Although the focus of this review is on tDCS, we would like to briefly acknowledge that other electric stimulation techniques, such as tACS and tRNS, seem to better preserve blinding integrity. tACS, which allows the entrainment of intrinsic brain oscillations through the administration of sinusoidal currents at specific frequencies [97], induces less side effects, such as muscle twitching, discomfort, and nausea [98], thus minimizing subjective sensations that could undermine blinding. Similarly, tRNS, which is based on the application of several frequencies within a normally distributed frequency spectrum [99], induces less noticeable skin sensations than tDCS, thus allowing for good blinding control [100].

The sham stimulation procedures described above are consistently present in NIBS studies, to allow blinding and to prevent participants “guessing” the type of stimulation. Conversely, a systematic assessment of participants’ expectations and beliefs about the effects of the stimulation on the brain and on performance has been largely neglected, despite the high risk that pure placebo- or nocebo-like mechanisms modulate the effects of NIBS. Positive or negative outcomes in performance could be due to the beliefs and expectations about the effects of the stimulation, the interaction between the researcher and the participant, increased or decreased negative emotions, and increased or decreased motivation. The probability of inducing a placebo or nocebo effect in NIBS trials is quite high, since these effects are typically stronger with devices than with pills [101,102]. Moreover, the mere act of applying the coil or the electrodes and delivering the magnetic or electric pulses could represent a psychosocial context conducive to the evocation of placebo or nocebo effects [34] (Table 2). As described in the following paragraphs, these modulations could be similarly present in the sham and in the active stimulation groups or conditions, thus undermining the possibility of drawing definitive conclusions from the collected data. Furthermore, these effects have been described in experimental studies as well as in clinical trials, both with TMS and with tDCS.

## 3. The Role of Expectation in Shaping the Effects of NIBS

### 3.1. The Role of Contextual Factors in Shaping Outcome

The following paragraph is devoted to reviewing the existing literature on placebo and nocebo effects in TMS and tDCS trials. It is important to point out that most of these studies are not targeted at test placebo/nocebo effects directly but aim instead to investigate the effectiveness of TMS and tDCS by comparing active vs. sham (placebo) stimulation protocols. Therefore, stating the presence of placebo/nocebo effects in these scenarios is based on post hoc consideration (e.g., through meta-analytical studies) of the effects that have been documented following sham stimulation, which, as commonly assumed, are ascribable to the positive expectations about a procedure/treatment that is believed to be effective.

It is important to point out that beyond these “simple” placebo/nocebo effects linked to the administration of an inert treatment/procedure, other broader contextual factors that pre-exist, or surround the clinical/experimental setting, may play a key role in shaping the magnitude of an individual’s response, independently of whether the treatment/procedure is active or sham. For example, the mere act of leaving home to join the study location (i.e., the laboratory or the clinic), as well as the contact with the operator or clinician, may, in themselves, elicit specific expectations even before an individual is exposed to the treatment/procedure. These expectations, in turn, may interfere with the specific expectations about the treatment/procedure at the point of receiving it, influencing outcomes accordingly. In addition, it is reasonable to assume that procedures requiring more complex rituals in terms of preparation and set-up (e.g., stimulation techniques, surgery, fMRI) could have an even stronger impact on individuals’ expectations, irrespective of whether these procedures are active or sham [103].

Overall, mounting evidence provides insights into how contextual factors such as the encounter with the physician/experimenter, the characteristics of the healthcare/laboratory setting, verbal, and non-verbal communication, as well as an individual’s preferences, personality traits, prior beliefs, and experiences, are all crucial determinants of outcome [104]. Importantly, these factors fall under the neuroscientific and “holistic” meaning of placebos, which moves beyond the concept of an inert treatment and refers to the effect of the psychosocial context surrounding the therapy [105,106], which has also been described as the “symbolic impact of medical treatment or the treatment setting” [107]. The influence of these “meta-placebo factors” may act synergistically with the specific expectations about the effects of a certain treatment or procedure and either boost or reduce the response. For example, clinical outcomes of evidence-based interventions can be shaped by contextual elements such as medical equipment, verbal or non-verbal communication by clinicians, and rituals associated with the treatment, through both psychological and neurobiological mechanisms [108].

That said, we would like to point out that the focus of this review is on discussing the impact of expectations and beliefs on the outcome of NIBS trials, without delving into the origin or nature of these expectations (e.g., specific expectations about the treatment/procedure or broader expectations linked to more non-specific contextual factors). However, throughout this review we mainly refer to placebo/nocebo effects in their broader contextual meaning (see Section 2.1.)

A ground-breaking path for future research could be that of teasing apart which specific placebo factors may more strongly affect participants’ expectations and outcome in NIBS trials.

### 3.2. Placebo and Nocebo Effects of TMS

Repetitive TMS (rTMS) is applied in many clinical trials [35,102] with the aim of testing its effectiveness for the treatment of psychiatric and neurological conditions and as a neuromodulation approach to enhance the therapeutic process. However, recent work has suggested that the specific effects of rTMS may be blurred by placebo effects, whereby active and sham rTMS induce the same effects on symptom relief [109]. This methodological conundrum emerges from different clinical trials, in which the placebo effect of sham TMS was estimated by comparing pre- versus post-intervention effects in sham TMS groups [110]. With respect to psychiatric disorders, clinical trials on depression, a mental and behavioral disorder which affects a person’s thoughts, behavior, motivation, feelings, and sense of well-being [111], report larger placebo responses when sham rTMS is administered concomitantly to an antidepressant drug, suggesting that the placebo response can be amplified when rTMS is used as an add-on therapy to drug administration (Table 2). Moreover, a recent meta-analysis by Razza et al. [36] found that the placebo response to sham rTMS in depressed patients was large and equivalent to that observed in the active rTMS group. The amount of placebo response for sham rTMS was consistent with the placebo response rate observed in pharmacological trials, whereas no difference was found comparing different protocols of sham rTMS (e.g., sham coil, 45°-angled coil, or 90°-angled coil, Table 2). These findings are in concert with another meta-analysis [37], in which a comparison between the placebo response of pharmacological trials with antidepressant drugs (escitalopram) and the placebo response of rTMS trials showed large placebo responses independently of the type of intervention (pharmacological or brain stimulation). These results fit well with previous work showing that the magnitude of the placebo response in the placebo arm of antidepressant trials is large [112] and may even explain 67% of the improvement in the active drug group [113].

Noteworthy findings have also been observed for schizophrenia, a mental disorder characterized by continuous or relapsing episodes of psychosis, hallucinations (typically hearing of voices), delusions, paranoia, and disorganized thinking [114]. In this respect, a meta-analysis on sham rTMS in schizophrenic patients found that 45°-angled coils over the left temporoparietal junction elicited a greater placebo response compared to other sham coils [38], suggesting that the type of sham rTMS might also have a role in inducing the placebo response. In this study, a meta-analytic method was applied to obtain a combined, weighted effect size (Hedges’ g). The mean weighted effect size of the placebo effect across 21 studies was g = 0.29, which is considered small. Importantly, this study highlighted two factors impacting on the placebo effect: the design of the study and the type of sham stimulation. Regarding the first factor, it was shown that parallel studies induce a placebo response with a medium effect size, while no consistent placebo response was found in crossover studies, highlighting the critical role of placebo in parallel designs. As to the second factor, when focusing on studies using parallel designs, a higher effect size was found with a 45°-tilted coil compared with a 90°-tilted coil or sham coil [38] (Table 2).

Finally, in other disorders, such as obsessive-compulsive disorder (OCD) [39,115], and primary insomnia [40], no significant differences in symptom relief were observed when comparing sham vs. active rTMS, hinting at a powerful placebo effect also in these psychiatric conditions (Table 2).

As to neurological disorders, studies on epilepsy estimating the placebo effect of sham rTMS reported a consistently low change in seizure frequency after sham rTMS, with a 2% median reduction at 2 weeks and no reduction at both 4 and 8 weeks. The reduction of seizure frequency was significantly greater for real rTMS than for sham. In fact, there is evidence of relatively weak placebo responses in epilepsy. This may be related to many factors, including mechanisms underlying seizures, patients’ decreased susceptibility to expectancy-related mechanisms, and natural cyclical fluctuation of seizure frequency [41,116] (Table 2). In Parkinson’s disease (PD), a neurodegenerative disorder characterized by motor and cognitive impairments, Okabe et al. [42] found that patients’ total and motor scores for the Unified Parkinson Disease Rating Scale improved to the same extent with both active rTMS over the motor cortex and the occipital cortex, and with sham stimulation (administered through electrodes fixed on the head to mimic the sensation induced by real rTMS). This benefit also extended to patients’ depression scores, which improved with rTMS over the motor cortex and sham stimulation in the same way (Table 2). Remarkably, the placebo effect of sham rTMS observed in PD also seems to concern patients with functional motor disorders, whereby no differences in symptom relief were observed when comparing rTMS to a control stimulation, suggesting that rTMS may mainly act through cognitive/expectancy mechanisms, rather than by actual cortical neuromodulation [43].

In chronic conditions, such as chronic pain, learning mechanisms seem to be crucial in shaping the magnitude of sham rTMS-induced placebo analgesia. Pain relief was found to be significantly enhanced when sham rTMS followed a session of successful active rTMS and worsened when it followed an unsuccessful rTMS session [44]. These findings suggest that unconscious learning mechanisms (i.e., prior exposure to a successful active treatment) could be key determinants of the placebo response. In chronic migraine, Conforto et al. [45] found that sham rTMS over the DLPFC for eight weeks induced a greater amelioration compared to active rTMS, with a decrease in the number of headache days greater than 50% in the sham group, suggesting a powerful placebo response. The authors reasoned that sham rTMS administered over the vertex could have led to a decrease in the number of pain attacks in patients with episodic migraine. This type of stimulation might have potentiated placebo analgesia by increasing patients’ expectations and by inducing dopamine release (Table 2). Similar results had previously been reported by Teepker et al. [46]. In line with this, Granato et al. [47] investigated whether high frequency rTMS over the DLPFC, combined with suggestions to avoid medication overuse, could impact on chronic migraine compared to sham rTMS. Results showed similar improvements in headache and headache-related disability in the active and sham rTMS groups, suggesting that placebo-related factors could have played a crucial role (Table 2).

Beyond clinical pain, in experimental pain, a landmark study by Krummenacher and colleagues [48] employed low-frequency active or sham rTMS over the DLPFC after expectation-induced placebo analgesia. In a heat-pain paradigm, the authors tested whether placebo analgesia would affect pain threshold and tolerance and if placebo analgesia would be suppressed following inhibition of DLPFC. It was found that sham rTMS, but not active rTMS, significantly increased pain threshold and tolerance in those participants who were cued that TMS was an effective device. Furthermore, pain indexes were suppressed following rTMS over DLPFC, suggesting a prominent role of this area in the placebo analgesia process. Importantly, this study was aimed at directly testing the placebo effect of rTMS via an a priori manipulation of subjects’ expectations on the effectiveness of the device.

Finally, there is evidence that TMS interventions may also induce nocebo effects. In this regard, a meta-analysis by Zis et al. [49], which included randomized placebo-controlled TMS trials in various neurological and psychiatric conditions, found that even in the absence of active stimulation, adverse effects could emerge when TMS was applied. Notwithstanding this, adverse effects are generally more likely to occur after active TMS interventions.

Taken together, these studies suggest that sham rTMS might induce both placebo and nocebo effects and should encourage the scientific community to implement more efficient control procedures.

### 3.3. Placebo and Nocebo Effects of tDCS

There is mounting evidence that subjects’ expectations interact with the effects of tDCS in modulating cognitive performance, as well as clinically relevant behaviors.

Studies in this field can be categorized into two types: some studies actively induced positive or negative expectations in combination with active or sham tDCS, whereas other studies considered the lack of difference between sham and active stimulation as evidence of a placebo effect. In a study by Rabipour et al. [50], expectations about the effects of tDCS were actively induced by means of information sheets. Participants received information about high or low effectiveness of tDCS on executive functions (i.e., working memory). Participants’ expectations and performance were assessed on three occasions: at baseline, after participants had read simple written messages about either high or low effectiveness of tDCS, and after a single session of anodal tDCS over the left DLPFC during working memory training. It was found that the information about the high or low effectiveness of tDCS induced respectively high and low expectations, as evidenced by increased or decreased expectation ratings. These patterns were present irrespective of the type of stimulation (sham or active). More importantly, the authors found an interaction between the stimulation protocol and expectation priming, with a worse performance when participants received low expectation priming combined with active stimulation and a better performance at the working memory task when they received high expectation priming, irrespective of the stimulation. The results suggested that active stimulation might have interfered with performance when low expectation priming was administered, while no effect was present in the case of high expectation priming, suggesting a possible benefit of high expectations over tDCS [50] (Table 2). Finally, participants who received high expectation priming reported more positive experience, greater enjoyment, engagement, motivation, and satisfaction. These findings were confirmed in a following study by the same group [51], in which expectations were manipulated (high vs. low) and tDCS was applied over the left or right motor cortex. Two tasks (i.e., the grooved pegboard test and a choice reaction time task) were performed at baseline and after tDCS. Results confirmed that expectation ratings increased after high priming and decreased after low priming. However, performance at the motor and cognitive tasks was unaffected by the expectation manipulation and by tDCS [51] (Table 2).

The placebo effects of sham tDCS have been described also for other cognitive functions, such as orientation discrimination [34]. While active tDCS over the primary visual cortex induced no significant effect on an orientation discrimination task, a significant improvement in performance was found in the sham tDCS group compared to the no-tDCS group. These results confirm a strong placebo effect induced by the montage itself. In addition, in the context of neurofeedback, a procedure in which brain signals are recorded, processed in real-time and fed back to the participant to improve motor functions, cognitive performance, emotion regulation and behavior [117], Kober et al. [118] used sham tDCS before neurofeedback training as an active placebo intervention (i.e., interventions whose pharmacological properties are not relevant for the purported condition but are administered for their psychological effect) to investigate whether the expectation of receiving brain stimulation interfered with the ability to self-regulate brain signals. Half of the participants received sham tDCS as a placebo intervention before neurofeedback, while the other half received no intervention before neurofeedback. It was found that most participants believed they had received active tDCS. Although this procedure did not affect subsequent neurofeedback performance, immediately after the placebo intervention, functional connectivity between frontal and parietal brain regions was increased in participants receiving sham tDCS. This is consistent with the literature showing that a placebo procedure increases brain connectivity between anterior and posterior brain areas [119]. The authors [118] suggested that the expectation of receiving brain stimulation could interfere with the ability to self-regulate the EEG sensorimotor rhythm, thus underlining the importance of controlling for participants’ expectations in neurofeedback interventions.

The impact of sham tDCS in evoking placebo effects has emerged also in clinically relevant domains, such as pain perception [120]. For example, Aslaksen and colleagues [52] demonstrated that participants who underwent a sham stimulation protocol displayed a significant reduction of perceived pain intensity compared to participants without electrodes on the scalp, suggesting again that the mere act of mounting the tDCS electrodes could induce placebo analgesia. Moreover, participants in the sham tDCS group responded similarly compared to individuals assigned to the real stimulation group. In patients with fibromyalgia, pain perception, and other disease-related symptoms, such as fatigue, mood disturbances, and sleep problems, were found to improve soon after treatment with tDCS and at 6 months follow-up, regardless of the type of stimulation (active or sham tDCS) [53].

Beyond pain, the effects of sham tDCS also extend to another clinically relevant domain, namely, motor performance. In this context, a recent study by Wang et al. [54] surveyed expectations about tDCS enhancement of motor performance through an online questionnaire and explored whether these expectations varied depending on prior tDCS experience or knowledge, sex, and age. The results showed high expectancy scores about the positive effects of tDCS in improving motor performance. Moreover, expectations were higher in females and younger adults when prior experience of stimulation was provided [54].

Finally, in the context of diet and eating behavior, Ray et al. [55] investigated the effects of tDCS on the extent of food craving and eating, while inducing in participants expectations of receiving either active or sham tDCS. It was found that expectations were able to reduce craving and eating after both sham and active tDCS, with no significant difference between interventions. Moreover, tDCS alone did not reduce food craving and eating, and no interaction was found between expectations and tDCS. Interestingly, the expectation alone yielded a 37.4% reduction in kcal consumed after only one session. This study indicated that induction of expectations coupled with tDCS montage (even in the absence of active stimulation) was effective in reducing kcal consumed (37.4% reduction after only one session).

The impact of the nocebo effect in tDCS trials has been investigated in a recent study [56]. Here, the focus was on how negative expectations about tDCS could affect feelings of agency and error processing. The authors found that when participants were verbally instructed to expect impaired cognitive performance when a sham tDCS was administered, they reported reduced feelings of agency compared with a control condition. Moreover, the expectation of impairment increased frontal theta power, potentially reflecting a process of increased cognitive control allocation, and stronger error-related negativity (ERN) was present when participants perceived tDCS efficacy, while it was absent when participants rated the efficacy as low. Interestingly, expectations impacted not only on the sense of agency, but also the EEG correlates.

## 4. Toward a Systematic Assessment of Participants’ Expectations in NIBS Studies

According to the evidence presented so far, the outcomes of NIBS can be affected by participants’ beliefs about the type of stimulation received and by the expectations and prior beliefs about the effects of the stimulation. These observations emphasize the importance of systematically assessing subjective expectation and beliefs in NIBS trials. For instance, between-groups designs present the important problem of whether the groups are balanced for expectation. If positive expectations about the effects of stimulation on performance are present in the sham group, but not in the active stimulation group, it is possible to find no advantage of the active stimulation over the sham, which could somehow “mask” the real effect of the stimulation. On the other hand, the presence of positive expectations in the active stimulation group, but not in the sham group, might result in a significant difference between groups due only to the different expectation levels and not to the intervention itself. In this framework, ensuring similar expectations in the two groups could allow the drawing of more definitive conclusions about the effectiveness of the treatment. An even more complex issue arises if we consider the interaction between positive and negative expectations and activating versus inhibiting stimulation protocols. This is particularly true if we assume a polarity-dependent effect of tDCS, whereby anodal tDCS is understood to enhance brain activity and cathodal tDCS is taken to reduce it [8]. Within this reference frame, when the protocol administered is thought to enhance the neural activation (e.g., through anodal tDCS), but the subjective expectations are low, it is possible to observe no modulation or even improved performance after sham. Conversely, when the protocol administered is thought to inhibit the activation (e.g., though cathodal tDCS), but the subjective expectations are high, we might observe improved performance, despite the type of stimulation administered. Finally, when expectations are consistent with the type of stimulation to be administered (e.g., anodal tDCS and high expectations, cathodal tDCS and low expectations), better or worse performance might be due to the combination of expectations and stimulation, and not only to the stimulation itself.

In within-subjects designs the critical issue is the possibility that participants could “guess” the stimulation applied by comparing the sensations felt on the skin during the stimulation sessions. However, it is possible, even in this case, that different expectations are present in the same subject during different stimulation sessions, depending on the participant’s emotions and mood at that moment, or in interaction with the researchers.

Although these considerations are critical for conducting reliable experiments, only a small number of NIBS studies have collected subjective information related to expectations [91]. To systematically assess participants’ expectations and beliefs in NIBS trials, two main approaches could be used: After the stimulation session, the experimenter could assess participants’ belief on the type of stimulation received (sham or active). For this, questionnaires are already available in the literature [121] and could be systematically applied in different studies. This information may also allow for stratification of participants based on their belief about the type of stimulation received with post hoc analysis of whether this belief had an impact on performance. Alternatively, participants’ expectations about the effects of the stimulation could be assessed prior to task performance. In this case, the focus is not on the type of stimulation applied, but rather on the expected effects on performance. This approach is more related to potential placebo and nocebo effects and to their interaction with the effects of the stimulation.

The implications related to the assessment of participants’ expectations are twofold. First, the possibility that participants “guess” the stimulation type is critical for the blinding procedure and might have crucial consequences in the interpretation of the collected data. In particular, the presence of positive expectations about the effects of stimulation during sham, but not active stimulation, might induce a failure in detecting a significant difference between the protocols, blurring the effect of real stimulation. Conversely, positive expectations about active stimulation, but not sham stimulation, might influence the results in the opposite way, with a possible greater enhancement after active stimulation due only to expectations, and not to the real stimulation per se. In these cases, expectations might act as an uncontrolled, confounding factor. This possibility is corroborated by experimental evidence: it was demonstrated that blinding issues are present both in sham TMS and in sham tDCS, with participants “guessing” the type of stimulation administered based on the different perceptual sensations experienced during the stimulation [32]. This is particularly problematic in within-subjects designs or when participants have already taken part in NIBS experiments. Moreover, it was demonstrated that participants’ expectations might be influenced by the mere act of positioning the coil or the electrodes on the scalp [34], thus making sham stimulation suitable for studying placebo effects in different domains. Another issue raised in recent investigations is the possible neurophysiological effects of weak intensity current in those sham protocols which apply mild but continuous stimulation [91]. This problem is present in both tDCS and TMS research, because some sham modalities, such as those consisting of active stimulation applied on cortical areas considered not involved in the modulation to be achieved, can still induce uncontrolled neurophysiological effects.

Second, the literature provides convincing evidence that it is possible to directly induce expectations in participants through active manipulation. Interestingly, some researchers have found an interaction between tDCS and the expectations that are experimentally induced [50], although investigations in this direction are still lacking. Shedding new light on these mechanisms might be crucial also in clinical applications and in experiments seeking to enhance the effects of brain stimulation. It remains to be investigated whether NIBS coupled with the induction of positive expectations about the interventions might result in a greater enhancement of cognitive functions or clinical outcomes, potentially making this manipulation an optimal strategy to achieve better results.

## 5. Concluding Remarks

It is now well established that NIBS has great potential in experimental and clinical research. For instance, NIBS has been shown to be effective in modulating fear memories and emotional processing and, therefore, could have a major impact in pathological conditions, such as those characterized by anxiety disorders [122,123,124]. Despite the great potential of NIBS in experimental and clinical research, concerns about the impact of subjective expectations and beliefs on results have recently been raised. Subjective expectations might induce either an improvement or a worsening in performance and modulate cortical activity as well, and they could interact with the stimulation or determine a failure in blinding procedure. To overcome these problems, we suggest a systematic evaluation of subjective expectations and beliefs before and after the stimulation and the collection of subjective predictions about the intervention. This information will be useful in ruling out or evidencing the possible interference of subjective expectations or predictions.

With regards to the potential mechanisms underlying the influence of expectations on stimulation effectiveness, we refer to the state-dependency concept [24]. According to this model, the effect of the magnetic pulse or the electric current on a brain area not only depends on the physical properties of the stimulus but also on the baseline activation state of that brain region. Using this framework, it is reasonable to hypothesize that subjective expectations could induce a particular brain state that in turn interacts with the effect of the magnetic or electrical stimulation. With respect to this, it is well established that the dorsolateral prefrontal cortex (dlPFC) is involved in elaborating expectations [48,125] and plays a prominent role, together with other brain regions, in the placebo effect [48,126,127,128,129,130,131,132,133]. Moreover, this associative brain area is involved in many higher-order cognitive functions, such as working memory [134,135], strategic planning ability [136], and attention [137], and plays a significant role in the executive top-down control of behavior [138]. Hence, we speculate that subjective expectations may change the activity of this brain region, generating brain states that interact with the effects of NIBS. This remains a speculative hypothesis that requires investigation.

## Figures and Tables

**Table 1:** Effectiveness of blinding procedures in sham protocols and possible differences in subjective sensations.

Article	Aim	Sample	Stimulation	Method & Outcomes	Main Results
Duecker & Sack 2013 [27]	Explore the effects of TMS clicking sound and skin sensations on visual target detection.	18 healthy volunteers.	Type: sham TMS Active: none Sham: identical to a real TMS coil with a magnetic shield reducing the effective magnetic field by 80% Intensity: 30% maximum SO Site: C3 and C4 Coil: figure-of-eight sham coil.	Within-subject design. Single pulse sham TMS to the right or left hemisphere at 300, 250, 200, 150, or 100 ms prior to target onset. As control, 80 trials without TMS. Outcome: RT at simple detection task.	Shorter RT when sham TMS preceded the target by 150, 200, and 250 ms; sham TMS ipsilateral to the target improved RT.
Duecker et al. 2013 [28]	Investigate the time-dependency and task-dependency of the effects of TMS clicking sound and sensations.	14 healthy volunteers.	Type: single-pulse TMS Active: 50% maximum SO Sham: identical to a real TMS coil at 30% maximum SO Coil: figure-of-eight Site: Vertex.	Within-subject design. Active or sham TMS during the tasks at 1 out of 9 TMS time windows (from −400 to +400 ms in steps of 100 ms) time-locked to stimulus onset, interleaved with no TMS trials. Outcomes: RT at a detection task and an angle judgment task.	Pre-stimulus TMS pulse increased the readiness to respond resulting in decreased reaction times. Post-stimulus TMS impaired task performance. This effect was specific for the detection task. No significant difference was found between active and sham TMS.
Flanagan et al. 2019 [29]	Determine whether iTBS could be distinguished from sham stimulation.	20 healthy volunteers (only women).	Type: iTBS Active: 600 pulses at 60%RMT Sham: sham coil < 0.3 T at 100% SO Coil: figure-of-eight coil Site: M1.	Crossover design. Two consecutive visits. Outcome: subjective reports on the type and the effects of stimulation (after 1 h from stimulation).	Prediction at chance level (55%) after active iTBS. Correct prediction after sham (74%). More accuracy at the second visit.
Turi et al. 2019 [30]	Assess the effectiveness of blinding in sham (fade-in, short-stimulation, fade-out) and active tDCS protocols.	192 healthy volunteers.	Type: anodal tDCS Active: 1 mA, 20 min Sham: 1 mA, 15 s, fade-in/out: 30 s Montage: anode over F3, cathode over right supraorbital region.	Between-subjects design. Sustained attention to response task during the stimulation (40 min duration); tDCS applied in the first 20 min. Outcome: assessment of blinding and discomfort.	Subjects could accurately guess they were receiving active tDCS when they actually did. More discomfort after active than sham tDCS.
Greinacher et al. 2019 [31]	Assess the time course of sham tDCS-blinding.	32 healthy volunteers.	Type: anodal tDCS Active: 1 mA, 10 min Sham: 1 mA, 20 s, fade-in/out: 30 s Montage: anode over C3, cathode over the right forehead.	Within-subjects design. Reaction time task before and during the stimulation. In blocks 2–4, sham-blinding probe questions were inserted every 30 s to assess blinding.	Difference in perception of itchiness between active and sham tDCS; participants correctly guessed above chance (78.1%) the session involving sham tDCS.
O’Connell et al. 2012 [32]	Investigate the effectiveness of sham tDCS blinding and the effects of previous exposure to sham or active stimulation.	96 healthy volunteers.	Type: anodal tDCS Active: 2 mA, 20 min, fade-in/out: 5 s Sham: switched off after 30 s Montage: anode over M1, cathode over the contralateral supraorbital region.	Crossover design; two separate sessions (active tDCS or sham). Word memory task before the stimulation. Judgement about the received stimulation (rating their confidence).	First session: 72% receiving active and 56% receiving sham, correctly judged the stimulation. Second session: 89% receiving active and 88% receiving sham guessed correctly. Confidence higher when they judged they had received active tDCS in the first session.
Brunoni et al. 2014 [33]	Compare blinding integrity and associated factors for tDCS vs. placebo-pill.	102 patients with major depression.	Type: tDCS Active: 2 mA, 30 min Sham: 2 mA, 30 s; fade-in 30 s; fade-out: 15 s Montage: anode over F3, cathode over F4.	Parallel design. Patients randomized to verum/placebo sertraline and active/sham tDCS. 10 sessions for the first 2 weeks, and 2 follow-up sessions every 2 weeks, for a total of 6 weeks. Outcome: assessment of blinding.	Both sertraline and tDCS mode were guessed above chance at week 6. Adverse effects and clinical response associated with correctly guessing.

TMS = transcranial magnetic stimulation; SO = stimulator output; RT = reaction times; iTBS = intermittent theta burst; tDCS = trascranial direct current stimulation.

**Table 2:** Placebo/nocebo effects in NIBS studies.

Article	Aim	Sample	Stimulation	Method & Outcomes	Main Results
Bin Dawood et al. 2019 [34]	Investigate the effects of occipital tDCS applied between two runs of orientation discrimination task (ODT).	Experiment 1: 66 healthy volunteers; experiment 2: 41 healthy volunteers.	Type: anodal and cathodal tDCS Active: 2 mA, 10 min Sham: 2 mA, 30 s, fade-in/out: none Montage: anode over Oz, cathode over left cheek and vice-versa.	Between-subjects design. Experiment 1: either anodal, cathodal, or sham tDCS. ODT administered at baseline and at the end of the stimulation. Experiment 2: baseline ODT, either no-tDCS with 2 min delay or 10 min delay between runs or receiving 10 min sham tDCS between the runs.	Experiment 1: Improvement in the second run of ODT compared to the first one regardless of the tDCS type. Experiment 2: only sham tDCS improved performance.
Hadi et al. 2020 [35]	Single case study of a patient with OCD and generalized anxiety disorder.	A man with anxiety symptoms and compulsive checking.	Type: 10 Hz rTMS Active: 2000 total pulses Intensity: 110% RMT Sham: none Site: lDLPFC.	rTMS during a 10-day period while taking no medication. Two additional sessions: one 6 months and one 8 months later.	Remission of symptoms after the first 10 days treatment, but also after the single session 6 months and 8 months later. Dramatic remission of symptoms after every rTMS session (even with single session rTMS) probably indicating a placebo effect.
Razza et al. 2018 [36]	Assess the magnitude of the placebo (sham) response to rTMS in major depressive disorder.	n.a.	n.a.	Meta-analysis of 61 studies.	Large placebo response directly associated with depression improvement of the active group, and inversely associated with higher levels of treatment-resistant depression.
Brunoni et al. 2009 [37]	Assess placebo responses in pharmacological (antidepressant drugs) and non-pharmacological (rTMS device) depressive disorder trials.	n.a.	n.a.	Meta-analysis of 41 studies: 29 in rTMS arm and 12 in the escitalopram arm.	Large placebo response for both escitalopram and rTMS studies. Sham response associated with refractoriness and with the use of rTMS as an add-on therapy.
Dollfus et al. 2016 [38]	Evaluate the placebo effect magnitude in rTMS treatment of auditory verbal hallucinations in schizophrenia, considering the type of sham used.	n.a.	n.a.	Meta-analysis of 21 randomized, double-masked, sham-controlled studies.	Significant effect size of sham in 13 parallel design studies, but not in the 8 crossover studies. Highest effect size observed with the use of the 45° position sham coil.
Mansur et al. 2011 [39]	Evaluate the efficacy of rTMS in patients with treatment-resistant OCD.	27 OCD patients.	Type: 10 Hz rTMS Active: 60,000 total pulses, 40 trains, 5 s per train Intensity: 110% RMT; ISI: 25 s Sham: deactivated TMS coil Coil: figure-of-eight coil Site: rDLPFC.	Between-subjects design (active rTMS: 13 patients; sham rTMS: 14 patients). Outcome: clinical improvement, depression, anxiety, and cognitive tests applied at baseline, after rTMS and at follow-up.	Patients were not able to discern group allocation during or after rTMS treatment. Questionnaires and cognitive tests were not affected by the group (active rTMS vs sham).
Jiang et al. 2019 [40]	Examine the efficacy and placebo response of rTMS in primary insomnia.	n.a.	n.a.	Meta-analysis of 9 clinical trials evaluating the efficacy of rTMS.	Active rTMS significantly improved insomnia symptoms for 10 days, 20 days, and 30 days. The proportion of sham rTMS response to the active rTMS response was 73.5%.
Bae et al. 2011 [41]	Investigate the sham rTMS placebo effect in epilepsy, comparing different coil positions.	n.a.	n.a.	Meta-analysis of 3 placebo-controlled rTMS trials in epilepsy. Three treatment conditions were studied: placebo-rTMS, target-rTMS (coil positioned over a cortical seizure focus), and nontarget rTMS (the coil not positioned over a cortical seizure focus).	Median seizure frequency was low and essentially unchanged by placebo rTMS, neither in post-treatment nor in follow-up.
Okabe et al. 2003 [42]	Investigate the efficacy of 0.2 Hz rTMS on Parkinson’s disease (PD) in comparison with sham.	85 patients with PD.	Type: 0.2 Hz rTMS Active: 110% AMT Sham: electrical stimulation on the head (0.2 msec, 2 times the sensory threshold) and coil over Cz to produce a sound. Coil: circular Sites: M1 (coil over Cz) and occipital cortex (coil over the inion).	Between-subjects design. Participants were randomly assigned to M1, occipital, and sham stimulation. Outcomes: subjective improvement, UPDRS scores, HRSD scores, measured at baseline, 4 weeks, and 8 weeks after treatment.	No difference in UPDRS and HRSD between groups; significant difference in subjective improvement between M1 and occipital stimulation (with better performance in the former).
Garcin et al. 2017 [43]	Investigate whether the positive effect of TMS in FMDs is due to cortical neuromodulation or to a cognitive-behavioral effect.	33 patients with FMDs.	Type: 0.25 Hz rTMS or 0.25 Hz RMS Active: 120–150% RMT Sham: none Site: lateral or medial motor cortex contralateral to symptoms for rTMS; cervical or lumbar spinal roots homolateral to symptoms.	Crossover design. Patients randomized to receive RMS on day 1 and rTMS on day 2 or vice versa. Outcomes: clinical assessment using a rating scale specific for FMDs. Follow-up at 3 days, 3 months, 6 months, and 1 year.	The median percentage of improvement was 29.2% after the first session and 18.2% after the second session. Similar improvement after RMS and TMS. On the third day, 60% of the patients were much or very much improved; at 1 year, 56% of patients were much or very much improved. No difference between the scores at the follow-ups.
Andrè-Obadia et al. 2011 [44]	Compare the analgesic effect of sham rTMS, either preceding or following active rTMS, in chronic pain.	45 patients with chronic neuropathic pain resistant to drugs.	Type: 20 Hz hf-rTMS Active: 20 trains of 80 pulses ISI: 84 s Intensity: 90% RMT Sham: sham coil Coil: figure-of-eight Site: M1.	Crossover design. 2 sessions of active and sham rTMS separated by 2 weeks. Outcomes: pain scores (5 days before the first session, after the first session, and continuously for 2 weeks).	Placebo analgesia differed significantly when the sham rTMS session followed a successful or an unsuccessful active rTMS. Placebo sessions induced significant analgesia when they followed a successful rTMS, whereas they tended to worsen pain when following an unsuccessful rTMS.
Conforto et al. 2014 [45]	Investigate the feasibility, safety, and efficacy of active rTMS in patients with chronic migraine without severe depression.	14 patients with chronic migraine (all women).	Type: 10 Hz rTMS Active: 1600 total pulses, 32 trains, 5 s per train Intensity: 110% RMT ISI: 30 s Sham: coil perpendicularly to the vertex Coil: figure-of-eight coil Site: DLPFC.	Parallel design. Active or sham rTMS in 23 sessions within 8 weeks. Outcome: feasibility, proportion of adverse events, number of headache days in the past four weeks (at baseline, after four and eight weeks).	No significant differences in compliance with the sessions of treatment between the sham and active groups; decrease in number of headache days in the sham group. No significant decrease in number of headache days and more perceived pain in the active group.
Teepker et al. 2010 [46]	Evaluate the therapeutic effects of low frequency rTMS in migraine.	27 patients with migraine.	Type: 1 Hz rTMS Active: 2 trains of 500 pulses, ISI: 1 min Intensity: able to produce a visually detectable muscle contraction in at least 5 out of 10 trials Sham: deactivated coil Coil: circular for active, figure-of-eight for sham Site: Vertex.	Parallel design. Active or sham rTMS in 5 consecutive days. Outcomes: reduction of migraine attacks, number, and hours of days with headache, pain intensity, and analgesic intake for migraine.	After active rTMS, a significant decrease in migraine attacks was observed, however, this effect was not significantly different from sham group. The same was true for days with migraine and total hours of migraine.
Granato et al. 2019 [47]	Investigate the effects hf-rTMS combined with suggestion to avoid medication overuse in patients suffering with chronic migraine and medication overuse headache.	14 patients with chronic migraine.	Type: 20 Hz hf-rTMS Active: 10 trains of 2 s duration ISI: 30 s Intensity: 100% RMT Sham: sham stimulator able to induce the same skin vibratory sensation Coil: circular for active, figure-of-eight for sham Site: DLPFC.	Parallel design. 14 patients assigned to active hf-TMS and 14 to sham. 5 consecutive days per week, for two weeks. Outcomes: headache duration and intensity, symptomatic drug intake, recorded at baseline and 1, 2, and 3 months after the first stimulation.	All outcomes decreased in the two groups without significant differences.
Krummenacher et al. 2010 [48]	Investigate the interaction of rTMS and expectations on pain perception.	40 healthy volunteers.	Type: 1 Hz hf-rTMS Active: 2 trains of 15 min each Intensity: 100% RMT Sham: sham coil Coil: figure-of-eight Site: F3 and F4.	Between-subjects design. Analgesia-expectation group (TMS as a painkiller) and control group (no effect of TMS on pain). Of these, half assigned to active TMS and half to sham TMS. Heat-pain paradigm, low-frequency rTMS or sham TMS before expectation-induced placebo analgesia.	Placebo significantly increased pain threshold and pain tolerance. rTMS treatment did not affect pain perception but the disruption of DLPFC activity with TMS completely blocked expectation-induced placebo analgesia. Analgesia-expectation group reported more effective pain reduction than the control group. Participants in the active-TMS group perceived less analgesic effect than those in the sham group.
Zis et al. 2020 [49]	Investigate the impact of nocebo phenomena during TMS clinical trials.	n.a.	n.a.	Meta-analysis of 93 placebo controlled randomized trials (depression: 28.0%, psychotic disorders: 19.4%, stroke: 12.9%, Parkinson’s disease: 7.5%, pain: 6.5%).	The pooled estimates of patients experiencing at least one adverse effect after active TMS and sham TMS was 29.3% and 13.6%. The odds of experiencing an adverse effect were 2.6 times higher in the active TMS group compared to sham. In depression, the nocebo adverse effects rate was 12.2%, while in depression the pooled estimates were 44.7% and 4.5%.
Rabipour et al. 2018 [50]	Investigate the potential influence of expectations on tDCS intervention outcomes.	90 healthy volunteers.	Type: anodal tDCS Active: 2 mA, 20 min Sham: 2 mA, 30 s, fade-in/out: 30 s for the single-blind round, 40 s for the double-blind round. Montage: anode over F3, cathode over supraorbital region.	Between-subjects design. High expectation priming (tDCS improves performance) or low expectation priming (tDCS has not known effects). Outcomes: expectations scores at baseline, after expectation priming and after tDCS; neuropsychological assessment; n-back task. Online task: working memory task.	Greater improvement in participants who received high compared to low expectation priming. Lowest performance after active tDCS and low expectation priming. Greater post-intervention improvement in executive function when receiving high compared to low expectation priming.
Rabipour et al. 2019 [51]	Investigate whether expectations could influence behavioral outcome of tDCS intervention.	121 healthy volunteers.	Type: anodal tDCS Active: 2 mA, 20 min Sham: fade-in/out: 30 s Montage: experiment 1 anode over M1 of the preferred hand, cathode over supraorbital region; experiment 2 anode over M1 of the non-preferred hand, cathode over supraorbital region.	Between-subjects design. High expectation priming (tDCS improves performance) or low expectations priming (tDCS has not known effects), and either active anodal or sham tDCS. Online task: finger fitness task. Outcomes: experiment 1: grooved pegboard test; experiment 2 grooved pegboard test, finger tapping test, and choice reaction time.	No significant effect in grooved pegboard test, in finger tapping test and choice reaction time in experiment 1 and 2. Participants primed to have high expectations significantly increased their expectation ratings compared to baseline, those who received low expectations priming significantly decreased their ratings.
Aslaksen et al. 2014 [52]	Investigate the effects of short-term tDCS on pain perception.	75 healthy volunteers.	Type: anodal tDCS Active: 2 mA, 7 min Sham: 2 mA, 30 s, fade-in/out: 20 s Montage: anode over C4 and cathode over the contralateral supraorbital area.	Between-subjects design. Three groups: active tDCS, sham tDCS, or no tDCS. Before, during and after tDCS painful stimuli were delivered (43, 45, 47 °C, for 20 s). Outcome: pain intensity, subjective stress, and blood pressure at baseline, after tDCS and in the post-test.	Pain decreased with active tDCS compared to no tDCS, no difference between active and sham at 45°. More effect of active tDCS compared to sham and no tDCS at 47°. More pain in the no tDCS group. Less subjective stress and lower blood pressure in the active tDCS compared to no tDCS group.
Samartin-Veiga et al. 2021 [53]	Establish the optimal area of tDCS stimulation in a sham-controlled trial in fibromyalgia.	130 healthy volunteers with fibromyalgia.	Type: anodal tDCS Active: 2 mA, 20 min, fade-in/out: 15 s Sham: fade-in/out: 15 s Montage: M1, DLPFC, OIC depending on the group.	Between-subjects design. Four groups: anodal tDCS over M1, DLPFC, OIC or sham. Outcome: pain intensity and improvement in other symptoms in fibromyalgia. 6 months follow-up.	Significant improvements across time for clinical pain and for fatigue, cognitive and sleep disturbances, and experimental pain, irrespective of the group. A significantly larger improvement after active tDCS, but not sham, in anxiety and depression.
Wang et al.2021 [54]	Assess expectations about tDCS as enhancer of motor performance and explore the role of prior experience and knowledge of tDCS, sex, and age.	379 healthy volunteers.	n.a.	Participants completed an online questionnaire through the Amazon Mechanical Turk (MTurk) platform.	Expectations about tDCS for improving motor performance were higher than neutral. Prior knowledge had larger influence on expectancy scores in females compared to males. Prior knowledge had large effect on expectancy scores among younger adults compared to older adults.
Ray et al. 2019 [55]	Evaluate the effect of tDCS on food craving and eating.	74 adults with body mass index ≥ 25.	Type: tDCS Active: 2 mA, 20 min Sham: 2 mA, 1 min at the beginning and at the end of the session, fade-in/out: none Montage: anode over F4 and cathode over F3.	Four groups: told fake/got fake, told fake/got real, told real/got fake, and told real/got real. Outcome: food craving task (how much they liked each food), eating task (kcal consumed) and hunger assessment.	Participants who were told they were receiving real tDCS craved less and ate significantly less kcals (37.4%) than participants who were told they were receiving fake tDCS. In both measures, no effect of real tDCS over sham was found.
Van Elk et al. 2020 [56]	Investigate how expectations about enhanced or impaired performance using tDCS affect feelings of agency and error processing.	57 healthy volunteers.	Type: tDCS Active: none Sham: 1 mA, fade-in: 1 min Montage: electrodes positioned over Afz and CPz.	Within-subjects design. Placebo condition: instructions about tDCS positive effect; nocebo condition: instructions about tDCS negative effect; neutral condition: no tDCS. Outcome: EEG and Eriksen flanker task. Subjective feeling of agency after errors, perceived efficacy, and suggestibility.	Better performance perception in the placebo compared to the nocebo condition. Highest feelings of agency over the performance in the control condition, and lowest in the impairment condition. During the induction phase, expecting impaired vs. enhanced performance increased frontal theta power.

tDCS = transcranial direct current stimulation ; rTMS = repetitive transcranial magnetic stimulation; OCD = obsessive-compulsive disorder; RMT = resting motor threshold; ISI = inter-stimulus interval; rDLPFC = right dorsolateral prefrontal cortex, lDLPFC = left dorsolateral prefrontal cortex; DLPFC = dorsolateral prefrontal cortex; hf-rTMS = high frequency transcranial magnetic stimulation; AMT = active motor threshold; UPDRS = Unified Parkinson Disease Rating Scale; HRSD = Hamilton Rating Scale for Depression; FMDs = functional motor disorders; RMS = root magnetic stimulation; OIC = operculo-insular cortex; M1 = motor cortex; EEG = electroencephalography; ERN = error-related negativity.
